# Absence of IL-17A in *Litomosoides sigmodontis*-infected mice influences worm development and drives elevated filarial-specific IFN-γ

**DOI:** 10.1007/s00436-018-5959-7

**Published:** 2018-06-22

**Authors:** Manuel Ritter, Vanessa Krupp, Katharina Wiszniewsky, Anna Wiszniewsky, Gnatoulma Katawa, Ruth S. E. Tamadaho, Achim Hoerauf, Laura E. Layland

**Affiliations:** 1 Institute of Medical Microbiology, Immunology and Parasitology (IMMIP), University Hospital of Bonn, Sigmund Freud Str. 25, 53127 Bonn, Germany; 2 Ecole Supérieure des Techniques Biologiques et Alimentaires, Université de Lomé, Lomé, Togo; 3 German Centre for Infection Research (DZIF), Partner Site, Bonn-Cologne, Bonn, Germany

**Keywords:** *Litomosoides sigmodontis*, IL-17A, Th17, IFN-γ, CCL17, Regulatory T cell, Immune-regulation

## Abstract

Lymphatic filariasis, onchocerciasis and loiasis are widespread neglected tropical diseases causing serious public health problems and impacting the socio-economic climate in endemic communities. More than 100 million people currently suffer from filarial infections but disease-related symptoms and infection-induced immune mechanisms are still ambiguous. Although most infected individuals have dominant Th2 and regulatory immune responses leading to a homeostatic regulated state, filarial-induced overt pathology like lymphedema, dermal pathologies or blindness can occur. Interestingly, besides dominant Th2 and regulatory T cell activation, increased Th17-induced immune responses were associated with filarial infection and overt helminth-induced pathology in humans. However, the immunological mechanisms of Th17 cells and the release of IL-17A during filarial infections remain unclear. To decipher the role of IL-17A during filarial infection, we naturally infected IL-17A^−/−^ and wildtype C57BL/6 mice with the rodent filariae *Litomosoides sigmodontis* and analysed parasite development and immune alterations. Our study reveals that infected IL-17A-deficient C57BL/6 mice present reduced worm burden on days 7 and 28 p.i. but had longer adult worms on day 28 p.i. in the thoracic cavity (TC), the site of infection. In addition, infiltration of CD4^+^ T cells, CD4^+^Foxp3^+^ regulatory T and functional CD4^+^Rorγt^+^pStat3^+^ Th17 cells in the TC was reduced in IL-17A-deficient mice accompanied by reduced eotaxin-1 and CCL17 levels. Furthermore, mediastinal lymph node cells isolated from IL-17A^−/−^ mice showed increased filarial-specific IFN-γ but not IL-4, IL-6, or IL-21 secretion. This study shows that Th17 signalling is important for host immune responses against filarial infection but appears to facilitate worm growth in those that reach the TC.

**Electronic supplementary material:**

The online version of this article (10.1007/s00436-018-5959-7) contains supplementary material, which is available to authorized users.

## Introduction

Factors that distinguish interleukin 17 (IL-17) CD4^+^ T cells from other Th populations include their regulation by the defined transcription factors: STAT3 (signal transducer and activator of transcription 3) and RORγτ (retinoic acid receptor-related orphan receptor-γτ) in mouse; the aryl hydrocarbon receptor; and the requirement of IL-1β, IL-6 and TGF-β (transforming growth factor β) for their development (Korn et al. 2009). However, further studies have shown that Th17 cells produce the cytokines IL-17A, IL-17F, IL-22, IL-21 and TNF-α and primarily promote immunity against extracellular pathogens like bacteria, fungi, helminth and protozoa infections (Korn et al. 2009; Eyerich et al. 2017). Moreover, IL-23 has been shown to be essential for the maintenance of Th17 responses in vivo (Korn et al. 2009*)* and the promotion of T-bet expression and thus inflammatory actions of Th17 cells (McAleer and Kolls 2011). In many settings, Th17 cells contribute to inflammation through the recruitment of neutrophils and instigate the release of pro-inflammatory mediators, chemokines and metalloproteinases (Korn et al. 2009; Eyerich et al. 2017).

In endemic communities, filarial infections in man remain a public health concern, and currently, 100 million individuals suffer from either lymphatic filariasis (LF), onchocerciasis or loasis, placing filariasis amongst the major causes of global morbidity (Klion and Nutman 2011; Ramaiah and Ottesen 2014; World Health Organization 2016). Filaria-like *Wuchereria bancrofti* modulate human immune responses so that most individuals carry numerous worms and present a homeostatic regulated state including elevated IL-10, TGF-β, regulatory T cells (Treg) and IgG4. Severe forms of the disease are associated with higher levels of IgE and IL-4 but low worm burden (Hoerauf et al. 2005; Adjobimey and Hoerauf 2010; Arndts et al. 2012). Interestingly, Th17 cells have been associated with helminth-induced overt pathology (Katawa et al. 2015) including elevated expression levels of Th17 cytokine family members (IL-17A, IL-17F, IL-21, IL-23) in peripheral blood mononuclear cells (PBMCs) from LF patients with chronic pathology (Babu et al. 2009). However, Metenou et al. also observed increased basal levels of IL-17A^+^CD4^+^ T cells in filarial-infected patients when compared to endemic normals (Metenou et al. 2010). Our previous studies have shown that PBMCs isolated from amicrofilaremic LF patients secrete more IL-17 levels upon activation with αCD3/αCD28 compared to endemic normals (Arndts et al. 2012) and moreover that Th17 cell frequencies are elevated in hyperreactive onchocerciasis and reduced in endemic normals accompanied with elevated CD4^+^IFN-γ^+^ frequencies (Katawa et al. 2015). Despite these observations, the immunological mechanisms associated with parasite control and disease progression in regard to Th17 signalling are not fully understood.

Thus, to further decipher the role of IL-17A in early phase of infection, we determined whether the lack of IL-17A during infection with *Litomosoides sigmodontis* altered parasite burden or immune responses like IFN-γ secretion in mice. This rodent filaria is employed to investigate many aspects of immune systems and pathways observed in human filarial infections (Hübner et al. 2009). Interestingly, *L. sigmodontis* infections in laboratory mouse strain are strain-specific since whereas infections in C57BL/6 mice are cleared after moulting into adult worms, BALB/c mice are fully permissive allowing the release of microfilariae, the worm’s offspring (Hübner et al. 2009). Within this study, we demonstrate that in the absence of IL-17A, infected IL-17A-deficient C57BL/6 mice present significantly reduced worm burden on days 7 and 28 p.i., but longer individual worms compared to C57BL/6 wildtype (WT) controls on day 28 p.i. Overall, IL-17A^−/−^ mice had reduced immune cell infiltration within the thoracic cavity (TC; the site of infection), especially reduced absolute cell numbers of CD4^+^ T cells, CD4^+^Foxp3^+^ Treg, CD4^+^Rorγt^+^pStat3^+^ and CD4^+^Rorγt^+^pStat3^+^ IL-17A^+^ Th17 cells. In regard to immune responses, eotaxin-1 and CCL17 levels in the TC of IL-17A-deficient mice were reduced, and moreover, mediastinal lymph node (mLN) cells isolated from IL-17A^−/−^ mice showed increased filarial-specific IFN-γ but not IL-4, IL-6 or IL-21 secretion and reduced Th17 cell frequencies. Taken together, this study investigates the role of IL-17A during *L. sigmodontis* infection and shows that IL-17A influences worm development through the dampening cellular responses in the site of infection and plays an important role in host immunity against this filarial nematode.

## Methods

### Animal maintenance, infections with *L. sigmodontis* and parasite recovery

Wildtype C57BL/6 and IL-17A-deficient C57BL/6 mice were bred at the IMMIP, University Hospital of Bonn, under SPF conditions in accordance with German animal protection laws and EU guidelines 2010/63/E4. Animal studies conducted in this manuscript were approved (84-02.04.2014.A301) by the local government authorities: Landesamt für Natur, Umwelt und Verbraucherschutz NRW, Germany. Verification of the genotyping was carried out using the following PCR primers: primer 1, 5′-ACTCTTCATCCACCTCACACGA-3′; primer 2, 5′-GCCATGATATAGACGTTGTGGC-3′; and primer 3, 5′-CAGCATCAGAGACTAGAAGGGA-3′. Primers 1 and 2 were used to detect wildtype allele (1.3 kb), and primers 1 and 3 were used to detect mutant allele (0.5 kb) (Nakae et al. 2002). The life cycle of *L. sigmodontis* was maintained in house using infected cotton rats and recovered adult worms were used as the source of *L. sigmodontis* antigen (LsAg) preparation (Rodriogo et al. 2016). Protein concentrations of antigenic extracts were determined using the Advanced Protein Assay (Cytoskeleton, ORT, USA) and aliquots of sterile LsAg were frozen at − 80 °C until required. Mice were infected using infected tropical mites as previously described (Hübner et al. 2009; Volkmann et al. 2003) and worms were recovered from the TC of individual mice 7 and 28 days p.i. to microscopically determine life stages, gender and length.

### TC lavage and cell differentiation analysis

Cytospins were prepared to analyse the composition of infiltrating immune cells within the TC fluid. To obtain TC cells, the cavity was rinsed with PBS and the resulting TC fluid was then centrifuged at 1200 rpm for 8 min at 4 °C. Cells were then used for flow cytometry analysis. Blood smears from individual mice were prepared using 5 μl of peripheral blood. Slides were then stained with Diff-Quick staining kit and cell populations were determined by analysing 100 cells/slide.

### Filarial-specific cell culture assays

For bulk cell assays, 5 × 10^5^ erythrocyte-depleted mLN cells from individual mice were plated in 96-well round-bottomed plates in RPMI 1640 medium containing 10% FCS, 1% Pen/Strep, 1% L-glutamine and 0.1% gentamycin (Thermo Fisher Scientific). Cells were left either unstimulated (Cont.) or stimulated with either αCD3/αCD28 (5 μg/1.25 μg/ml; eBiocience, Frankfurt, Germany) or LsAg (50 μg/ml) for 72 h at 37 °C. Supernatants were then removed and analysed for chemokines and cytokines.

### Cytokine and chemokine determination

Cell culture supernatants and TC fluid were analysed for cytokine and chemokine levels by ELISA according to the manufacturer’s instructions (IL-6, RANTES, granzyme B, eotaxin-1, CCL17: R&D systems, Wiesbaden-Nordenstadt, Germany; IL-4: BD, Heidelberg, Germany; IFN-γ, IL-21: eBioscience). ELISA plates were read and analysed at 450 and 570 nm using a Spectra Max 340pc384 photometer and SOFTmax Pro 3.0 software (Molecular Devices, Sunnyvale, CA, USA).

### Flow cytometry staining

Absolute cell counts from the mLN and TC of individual mice were determined using the CASY® Cell Counter and Analyser System Model TT (Roche Innovatis AG, Reutlingen, Germany). FACS staining was performed as previously described (Rodriogo et al. 2016). In brief, cells were fixed and permeabilised with intracellular fixation and permeabilisation buffer set (Thermo Fisher Scientific) according to the manufacturer’s instructions. Thereafter, cells were stained with combinations of fluorophores (FITC, PE, PE-Cy7, PerCp-Cy5.5, APC) conjugated with anti-mouse CD4, CD11b, Foxp3, F4/80, GR1, Ly6c, pStat3 (Y705), Rorγt (eBioscience) and IL-17A (Biolegend, San Diego, USA) monoclonal antibodies to determine distinct cell populations. Expression levels were determined using the FACS Canto flow cytometer (BD Bioscience) and analysed with FlowJo v10 software (FlowJo, LLC, USA).

### Statistical analysis

Statistical differences were determined using the software SPSS (IBM SPSS Statistics 22; Armonk, NY) and GraphPad Prism 5 (GraphPad Software, Inc., San Diego, CA, USA). Statistical significances between two groups were analysed using unpaired *t* tests or Mann-Whitney *U* test and comparison between more than two groups was analysed using ANOVA or Kruskal-Wallis tests for parametrically and non-parametrically distributed data, respectively.

#### Data availability

The datasets used and/or analysed during the current study are available from the corresponding author upon reasonable request.

## Results

### *L. sigmodontis*-infected IL-17A-deficient C57BL/6 mice have reduced worm burden

**Fig. 1:**
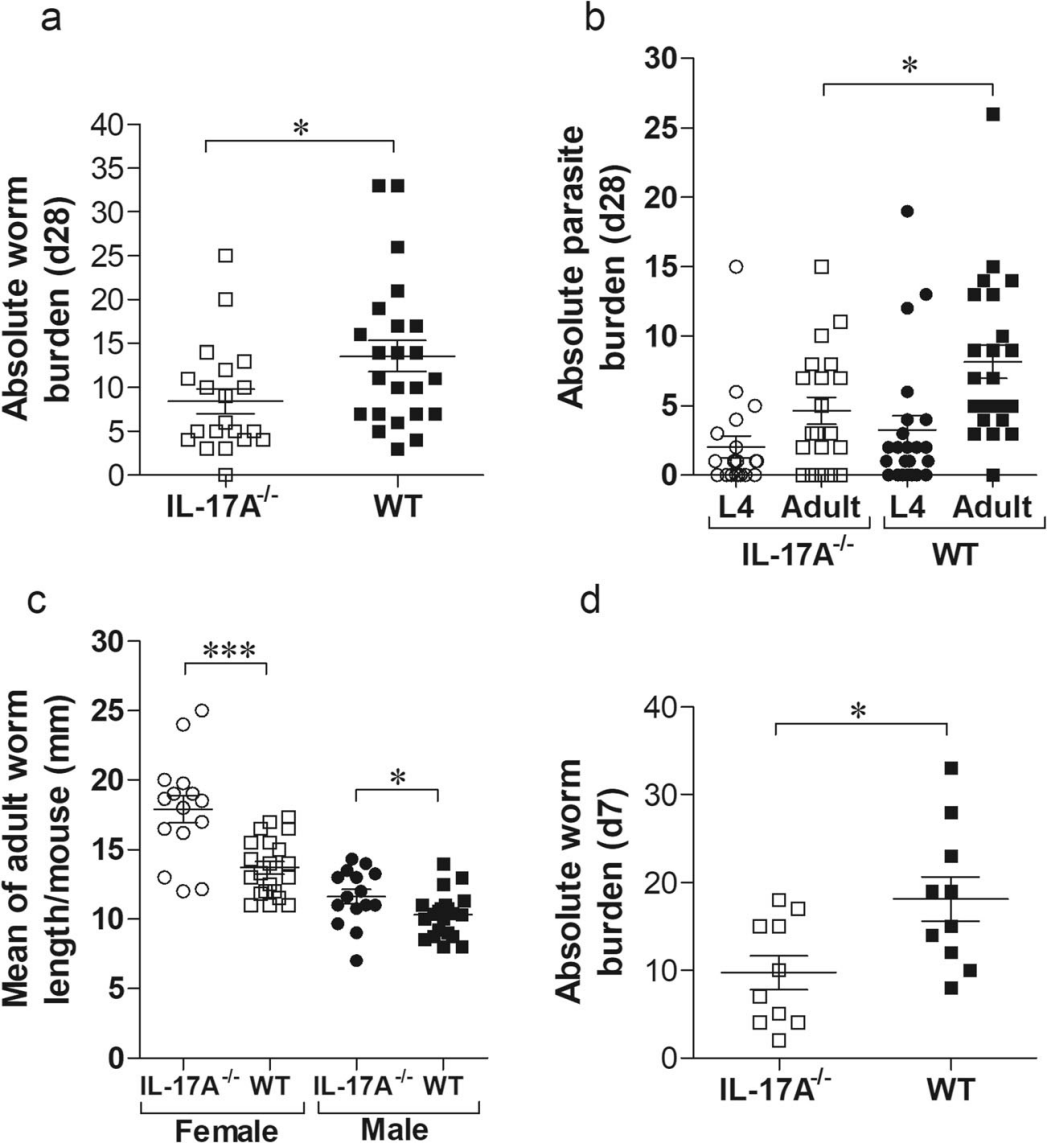
Reduced worm burden in *L. sigmodontis*-infected IL-17A^−/−^ C57BL/6 mice. Groups of WT and IL-17A^−/−^ C57BL/6 mice were infected with *L. sigmodontis* for 28 days (d28). Thereafter, absolute worm burden (**a**), life stage (**b**) and adult worm length (**c**) were determined in individual mice. Values are expressed as mean ± SEM from four independent infection experiments (*n* = 20 IL-17A^−/−^ and *n* = 23 WT mice). Data in **c** show mean ± SEM of adult worm length per mouse from the total *n* = 53 female and *n* = 38 male adult worms isolated from IL-17A^−/−^ mice and *n* = 70 female and *n* = 107 male adult worms isolated from WT mice. Values in **d** show absolute worm burden as mean ± SEM from groups of WT (*n* = 10) and IL-17A^−/−^ C57BL/6 mice (*n* = 10) on day 7 p.i. (d7). Statistical significances between the indicated groups were obtained using Mann-Whitney *U* tests. Asterisks denote significant differences between the groups indicated by the brackets (*p < 0.05, ***p < 0.001)

To assess whether the lack of IL-17A during infection altered worm burden and development in C57BL/6 mice, worm numbers and life stages were determined in IL-17A-deficient mice on day 28 p.i. This time point corresponds to the final moulting of L4 into adult worms (Hübner et al. 2009). In general, no anatomopathological differences could be observed between WT and IL-17A-deficient mice but IL-17A^−/−^ mice had a significantly lower worm burden than the infected WT group (Fig. 1a). This was reflected in the significantly reduced number of adult worms in the IL-17A^−/−^ mice (Fig. 1b). Worm burden analysis includes differentiating motile free-living worms and worms already encapsulated by host immune cells which form nodules—“granuloma-like structures”. However, 43.5% of WT and 40% of IL-17A^−/−^ mice had nodules, and in addition, female to male ratio was also equal between IL-17^−/−^ and WT mice. Nevertheless, adult female and male worms were significantly longer in IL-17A-deficient mice than adult worms recovered from WT infected mice (Fig. 1c). These data provide an indication that the absence of IL-17A supports worm clearance in the early phase of infection but also promotes worm growth in the TC. Therefore, to investigate the influence of IL-17A on an earlier time point, we analysed worm burden on day 7 p.i. since this time point corresponds to the migration of L3 larvae into the TC and the onset of moulting to L4 stages (Hübner et al. 2009). Indeed, IL-17A-deficient mice have again significantly lower worm burden compared to WT mice (Fig. 1d), suggesting that the lack of IL-17A supports worm clearance during the migration of L3 larvae through the skin into the coelomic cavities.

### Comparable absolute cell numbers of monocytes, macrophages, neutrophils and eosinophils within the TC

**Fig. 2:**
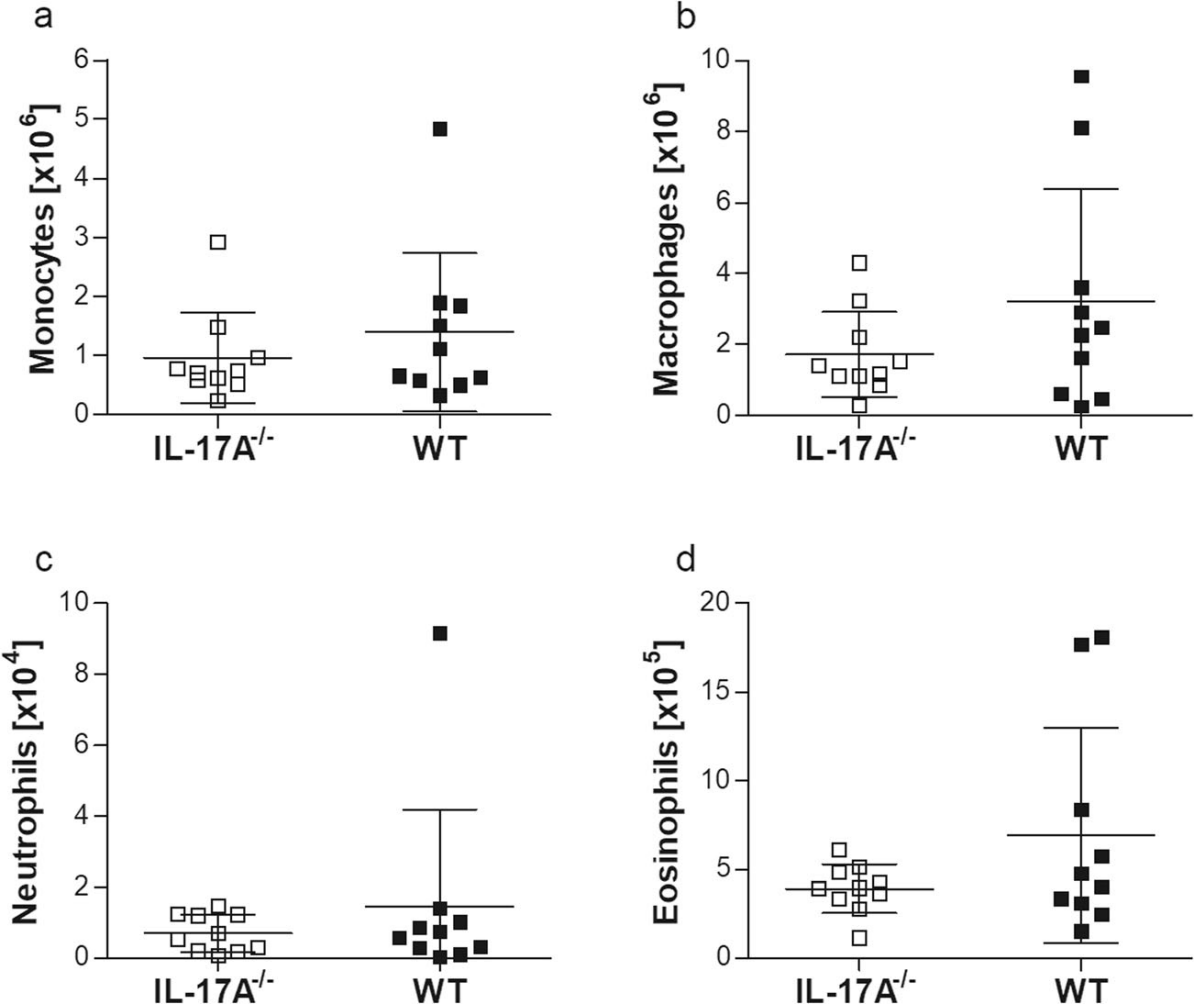
Flow cytometry-based differentiation of TC cells revealed comparable monocyte, macrophage, neutrophil and eosinophil numbers. Groups of WT and IL-17A^−/−^ C57BL/6 mice were infected with *L. sigmodontis* for 28 days. Within the TC, the site of infection, the absolute number of CD11b^+^SiglecF^−^Ly6c^+^ monocytes (**a**), CD11b^+^SiglecF^−^F4/80^+^ macrophages (**b**), CD11b^+^SiglecF^−^GR1^+^ neutrophils (**c**) and CD11b^+^SiglecF^+^ eosinophils (**d**) was determined in individual mice using flow cytometry. Values are expressed as mean ± SEM and symbols show levels in each mouse from one infection experiments (*n* = 10 IL-17A^−/−^ and *n* = 10 WT mice). Statistical significances between the indicated groups were obtained using the Mann-Whitney *U* tests

**Fig. 3:**
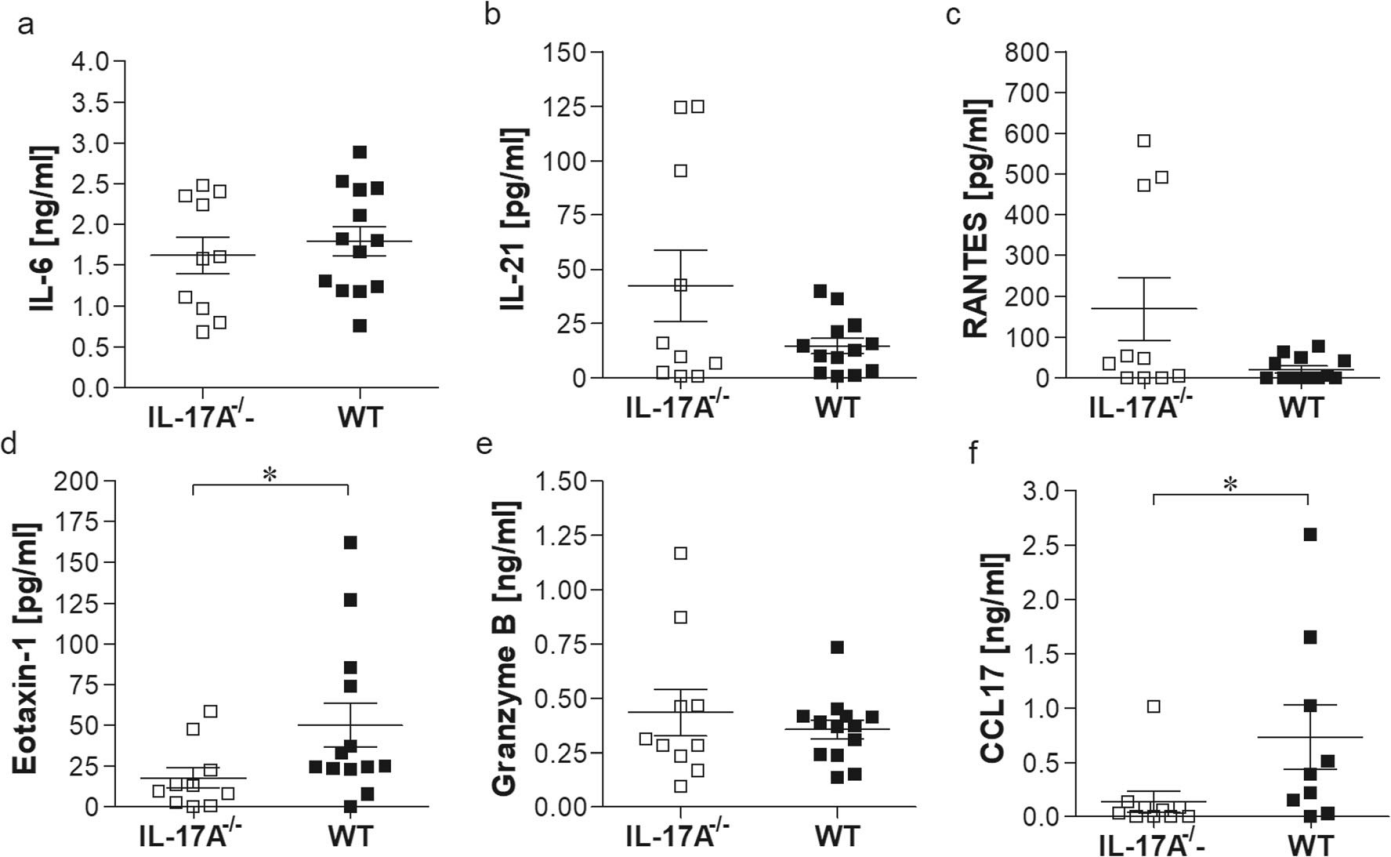
Reduced eotaxin-1 and CCL17 levels in *L. sigmodontis*-infected IL-17A^−/−^ C57BL/6 mice. Groups of WT and IL-17A^−/−^ C57BL/6 mice were infected with *L. sigmodontis* for 28 days. Levels of IL-6 (**a**), IL-21 (**b**), RANTES (**c**), eotaxin (**d**), granzyme B (**e**) and CCL17 (**f**) were determined in the lavage fluid from the TC by ELISA. Values are expressed as mean ± SEM and symbols show levels in each mouse from three independent infection experiments (*n* = 10 IL-17A^−/−^ and *n* = 13 WT mice). Statistical significances between the indicated groups were obtained using the Mann-Whitney *U* tests. Asterisks denote significant differences between the groups indicated by the brackets (*p < 0.05)

Next, we studied changes in the TC, the site of *L. sigmodontis* infection. Upon analysis of absolute cell numbers, infected IL-17A^−/−^ mice had a significantly reduced influx of immune cells when compared to WT mice (Online Resource 1a). To further differentiate immune cells within the TC, flow cytometry was performed and absolute numbers of monocytes, macrophages, neutrophils and eosinophils (Fig. 2) were analysed according to the applied gating strategy (Online Resource 2). Flow cytometry-based analysis of the infiltrating immune cells at the site of infection revealed comparable absolute cell numbers of monocytes (Fig. 2a), macrophages (Fig. 2b), neutrophils (Fig. 2c) and eosinophils (Fig. 2d). In blood, the frequency of cell populations was also comparable between both knockout and wildtype infected animals (Online Resource 3). Next, we measured parameters in the lavage fluid from the TC of individually infected mice which were previously shown to be important for Th17 development (IL-6, IL21 and IL-23) or *L. sigmodontis* infection (RANTES, eotaxin-1, granzyme B, CCL17) per se. IL-6 (Fig. 3a), IL-21 (Fig. 3b) and CCL5/RANTES (regulated on activation, normal T cell expressed and secreted; Fig. 3c) levels were comparable between infected IL-17A^−/−^ and WT mice. Levels of CCL11/eotaxin-1 were significantly increased in infected WT controls (Fig. 3d), despite the fact that eosinophil numbers in the TC were comparable between the two infected groups of mice (Fig. 2d), confirming previous results which showed that eosinophil migration to the site of infection is independent of eotaxin-1 secretion (Gentil et al. 2014). Levels of granzyme B were equal between infected WT and IL-17A^−/−^ mice (Fig. 3e) and no IL-23 could be detected in either group. In addition, CCL17 levels were significantly reduced in infected IL-17A^−/−^ mice compared to WT controls (Fig. 3f).

### *L. sigmodontis*-infected IL-17A-deficient mice present dominant filarial-specific IFN-γ responses

**Fig. 4:**
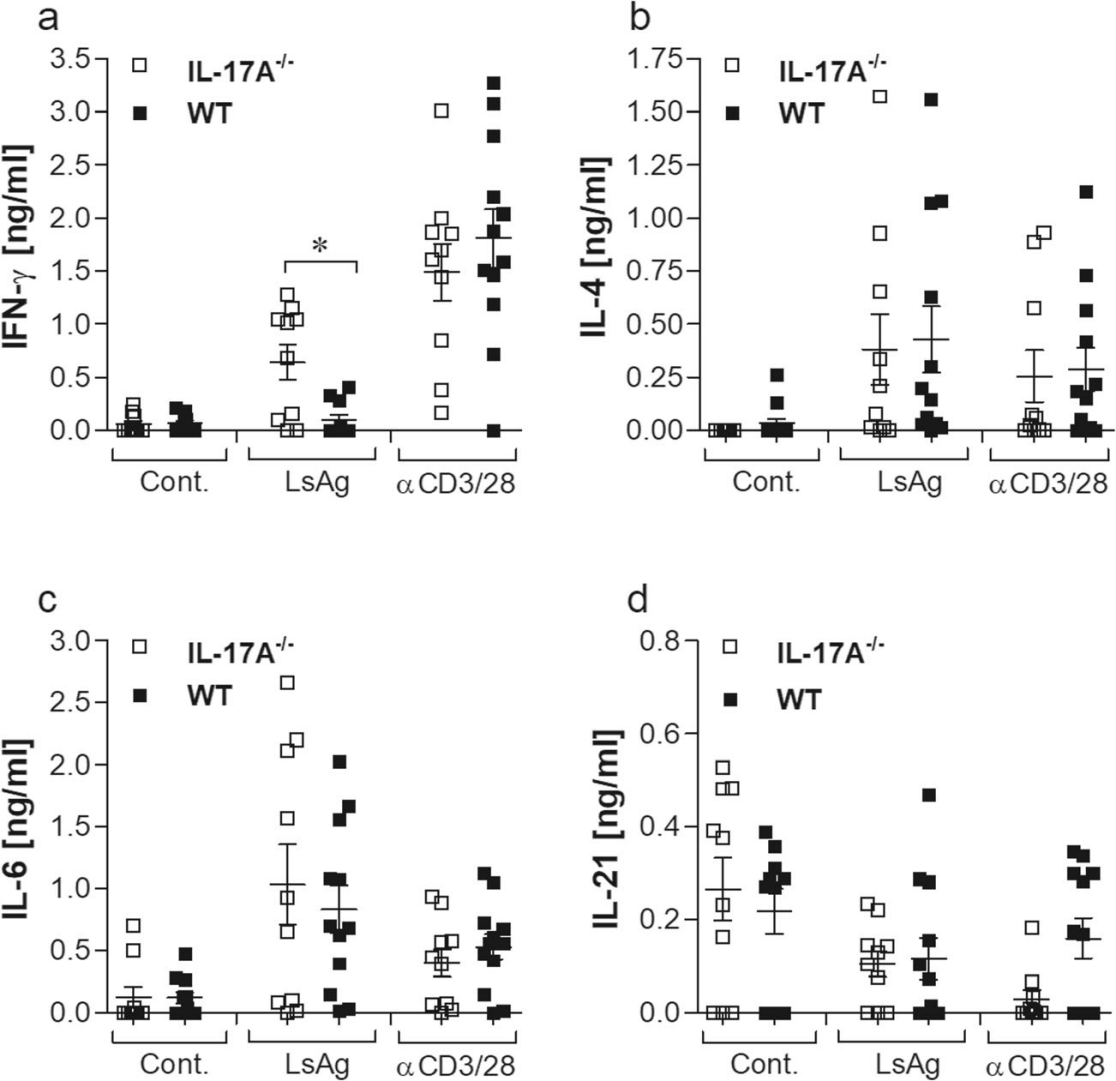
Infected IL-17A^−/−^ mice show dominant filarial-specific IFN-γ responses. On day 28 p.i., draining mLN cells (5 × 10^5^ cells/well) from individual mice were plated in RPMI 1640 medium with supplements and left either alone (Cont.) or stimulated with LsAg (50 μg/ml) or αCD3/αCD28 (5/1.25 μg/ml) in triplicates. After 72 h, the culture supernatant was removed and screened for the presence of IFN-γ (**a**), IL-4 (**b**), IL-6 (**c**) and IL-21 (**d**) by ELISA. Graphs show cytokine responses and values are expressed as mean ± SEM from each mouse from three independent infection experiments (*n* = 10 IL-17A^−/−^ and *n* = 13 WT mice). Statistical significances between the indicated groups were obtained using the Mann-Whitney *U* tests. Asterisks denote significant differences between the groups indicated by the brackets (*p < 0.05)

To determine whether the lack of IL-17A altered filarial-specific recall, cell cultures were prepared from mediastinal lymph nodes (mLN) from infected mice and either left alone (Cont.) or re-stimulated for 72 h with LsAg: an antigen source prepared from adult *L. sigmodontis* worms. Thereafter, the resulting supernatant was tested for levels of IFN-γ (Fig. 4a), IL-4 (Fig. 4b), IL-6 (Fig. 4c) and IL-21 (Fig. 4d). In general, cytokine levels were increased upon LsAg or αCD3/αCD28 (TCR activator) stimulation (Figs. 4a–c), except IL-21 responses which were reduced from basal levels in infected IL-17A^−/−^ and WT mice upon re-stimulation (Fig. 4d). Interestingly, as shown in Fig. 4a, infected IL-17A^−/−^ mice produced significantly higher levels of IFN-γ than WT infected mice upon LsAg but not αCD3/αCD28 re-stimulation, strongly indicating that IL-17A-mediated responses could inhibit filarial-specific IFN-γ secretion in the draining lymph nodes.

### Reduced CD4^+^Foxp3^+^ Treg at the site of infection in *L. sigmodontis-*infected IL-17A^−/−^ mice

**Fig. 5:**
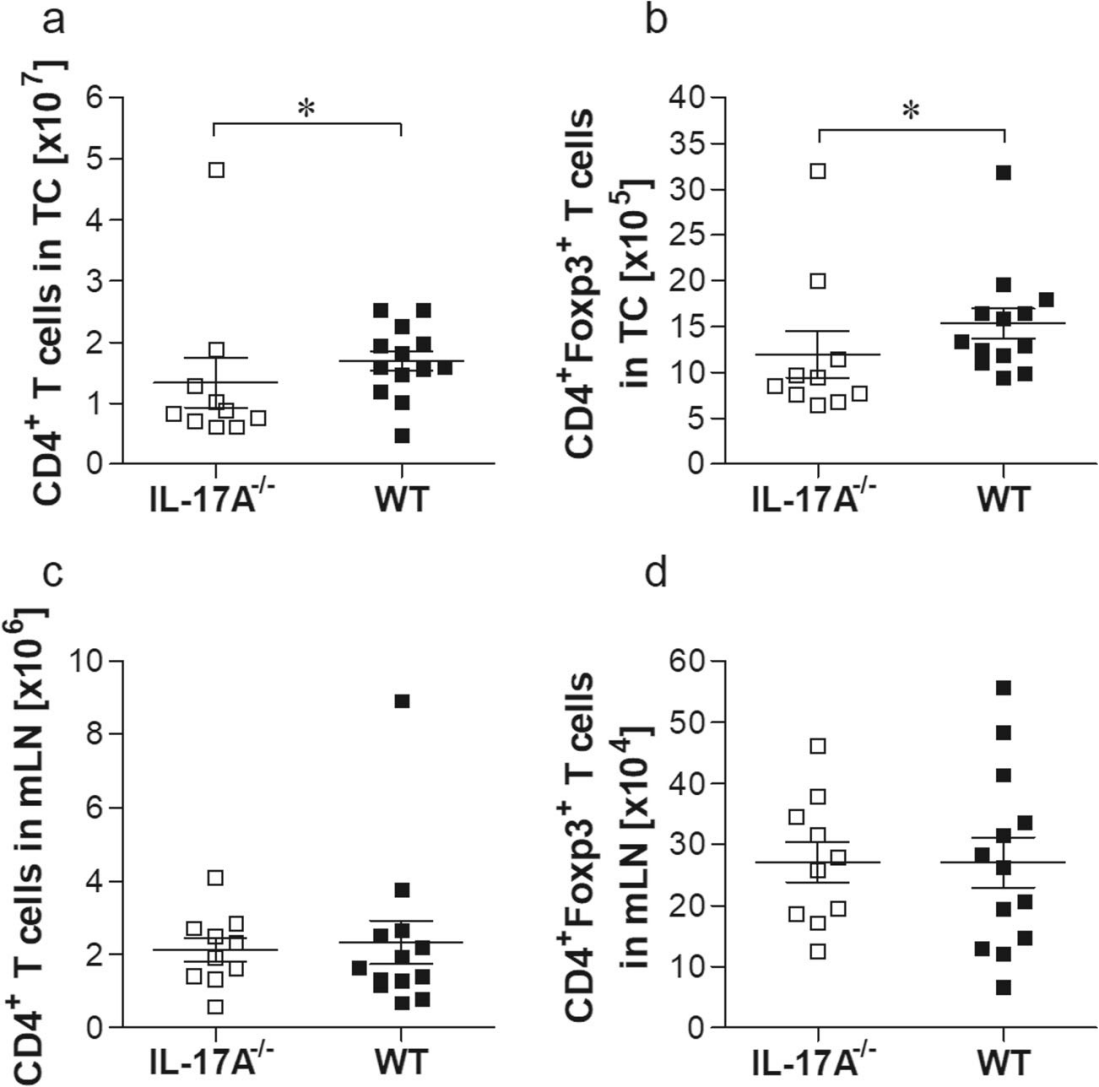
Reduced CD4^+^Foxp3^+^ Treg in *L. sigmodontis*-infected IL-17A^−/−^ C57BL/6 mice at the site of infection. On day 28 of infection, thoracic (**a**, **b**) and mLN (**c**, **d**) cell populations were screened for levels of CD4^+^ T cells (**a**, **c**) and CD4^+^Foxp3^+^ Treg (**b**, **d**) populations. Values are expressed as mean ± SEM and symbols show levels in each mouse from three independent infection experiments (*n* = 10 IL-17A^−/−^ and *n* = 13 WT mice). Statistical significances between the indicated groups were obtained using the Mann-Whitney *U* tests. Asterisks denote significant differences between the groups indicated by the brackets (*p < 0.05)

Several studies, including our recent study in onchocerciasis, have shown a relationship and interplay between Treg and Th17 cells which are important during inflammatory responses (Katawa et al. 2015; Weaver and Hatton 2009). Thus, we identified whether populations of CD4^+^ T cells and Foxp3^+^ Tregs within the TC (Fig. 5a, b) and mLN (Fig. 5c, d) altered upon infection according to the applied gating strategy (Online Resource 4). As mentioned above, infected IL-17A^−/−^ mice had an overall reduced number of cells within the TC (Online Resource 1a). This was not observed in cell counts of the mLN (Online Resource 1b). Whereas CD4^+^ T cell numbers were reduced in the TC of IL-17A^−/−^ mice (Fig. 5a), no changes were observed in mLN populations (Fig. 5c). The amount of Foxp3^+^ T cells within the CD4^+^ T cell compartment was also reduced in IL-17A-deficient mice (Fig. 5b), but again, this was not reflected in the mLN (Fig. 5d). These data indicate that whereas expansion of CD4^+^ T cells and Treg populations in the mLN is unaffected, infiltration of these T cell subsets into the TC is reduced in infected IL-17A^−/−^ mice.

### Th17 cells are reduced in *L. sigmodontis-*infected IL-17A^−/−^ mice

**Fig. 6:**
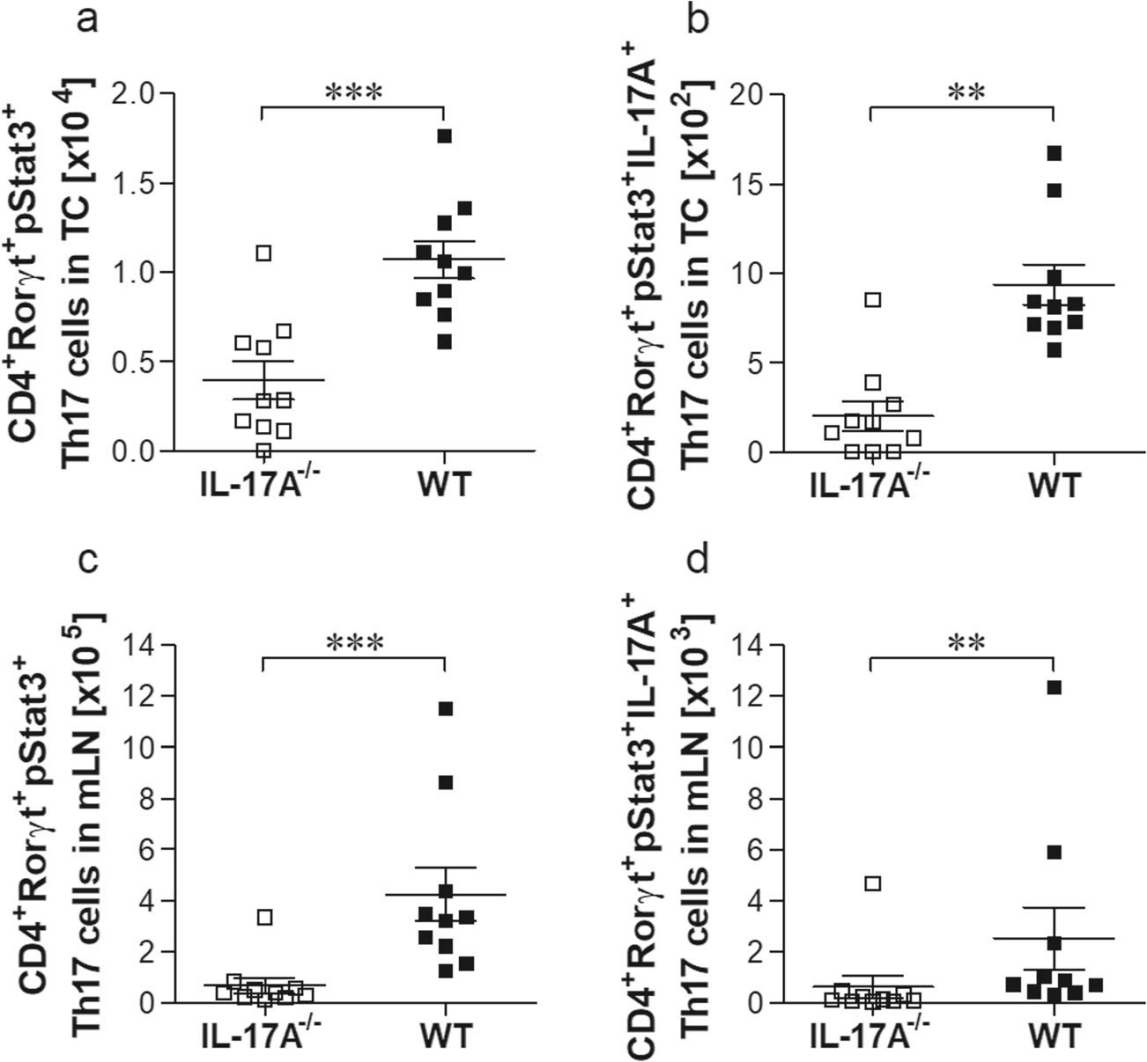
Impaired functional Th17 cells in *L. sigmodontis*-infected IL-17A^−/−^ C57BL/6 mice at the site of infection and the draining lymph nodes. On day 28 of infection, thoracic (**a**, **b**) and mLN (**c**, **d**) cell populations were screened for levels of CD4^+^Rorγt^+^pStat3^+^ (**a**, **c**) and CD4^+^Rorγt^+^pStat3^+^IL-17A^+^ Th17 cell (**b**, **d**) cell populations. Values are expressed as mean ± SEM and symbols show levels in each mouse from one infection experiments (*n* = 10 IL-17A^−/−^ and *n* = 10 WT mice). Statistical significances between the indicated groups were obtained using either the unpaired *t* test or the Mann-Whitney *U* tests. Asterisks denote significant differences between the groups indicated by the brackets (**p < 0.01 and ***p < 0.001)

Since the lack of IL-17A leads to enlarged worms concomitant with reduced Foxp3^+^ Treg at the site of infection, we further analysed Th17 cell populations in the TC and mLN on day 28 p.i. using flow cytometry according to the applied gating strategy (Online Resource 5). Indeed, absolute cell numbers of CD4^+^Rorγt^+^pStat3^+^ were significantly reduced (Fig. 6a) and almost no IL-17A-producing Th17 cells could be obtained in the TC of IL-17A-deficient mice (Fig. 6b). The significant reduction of functional Th17 cells was reflected in the mLN (Fig. 6c, d). In summary, the data suggested that the lack of IL-17A affects L3 migration into the TC and creates an environment which promotes worm development and growth at the site of infection.

## Discussion

Genetics (Debrah et al. 2011) and filarial-driven modulation of the host’s immune system, mainly through the maintenance of dominant Th2 immune responses and induction of Treg, are factors that are considered to control the host-regulated response to filarial infections (Hoerauf et al. 2005; Adjobimey and Hoerauf 2010; Arndts et al. 2012; Katawa et al. 2015). The underlying mechanisms maintaining such filarial-mediated immune homeostasis remain unclear and little data exists on why a small proportion of infected individuals develop overt pathology and filarial-related diseases. Since the identification of Th17 cells, studies have linked their activities to filarial infections. Babu and colleagues reported that Th17-associated cytokines like IL-17A were increased in PBMCs from patients with lymphedema upon *Brugia malayi* antigen stimulation (Babu et al. 2009). Increased CD4^+^IL-17A^+^ basal levels in filarial-infected (*Mansonella perstans* and/or *Wuchereria bancrofti*) patients from Mali (Metenou et al. 2010) and increased IL-17A secretion from PBMCs derived from microfilaria-positive LF patients upon αCD3/αCD28 stimulation (Arndts et al. 2012) imply a critical role of Th17 immune responses during LF infection. Further studies on onchocerciasis have linked an accentuated Th2/Th17 profile with individuals presenting severe forms of dermal pathology (Katawa et al. 2015) and higher IL-17A responses to a *Plasmodium-*derived antigen were observed in cell cultures from microfilaridermic individuals (Arndts et al. 2015). To further decipher the role of IL-17A on worm development in early phase of infection, we employed the rodent model of filariasis in C57BL/6 mice and demonstrate that infected IL-17A-deficient C57BL/6 mice develop longer but reduced numbers of adult worms and cell cultures secrete increased levels of filarial-specific IFN-γ. Further analysis showed that eotaxin-1 levels as well as infiltrating CD4^+^Foxp3^+^ Treg and CD4^+^Rorγt^+^pStat3^+^ Th17 cells were reduced in the TC. The reduced worm burden on days 7 and 28 p.i. concomitant with longer individual worm lengths on day 28 p.i. implies that the absence of IL-17A supports worm clearance in the early phase of infection but also promotes worm growth in the TC. Interestingly, *L. sigmodontis* infections in RAG2IL-2Rγ^−/−^ C57BL/6 mice that are deficient for T, B and natural killer cells (NK) result in persistently high numbers of adult worms which were significantly longer compared to C57BL/6 mice (Layland et al. 2015), showing that anti-filarial immune responses within the TC are crucial for parasite clearance. Therefore, we suggest that the improved growth of *L. sigmodontis* worms in the IL-17A^−/−^ mice is driven by the lack of IL-17A and not a result of the reduced competition for space and nutrients. In addition, no free-living worms or microfilariae could be observed in IL-17A-deficient mice on day 72 p.i. (data not shown), confirming that patency development in C57BL/6 mice depends on multiple immune cells (Layland et al. 2015).

It was shown that in humans with atopic dermatitis, a decreased circulation of Th17 (IL-17^+^) cells correlated with increased levels of CCL17 (Hayashida et al. 2010). Our group has previously shown that CCL17 controls mast cell activation and mast cell-dependent vascular permeability in the skin which is critical for the defence against invading L3 larvae during a *L. sigmodontis* infection (Specht et al. 2011). Moreover, using a mouse model for human allergic contact dermatitis, it was shown that IL-17 deficiency decreases CCL17 expression (Nakajima et al. 2014). In this study, we could show that CCL17 levels were reduced in TC fluid of IL-17A^−/−^ mice. In addition, high CCL17 expression in human PBMCs from *Onchocerca volvulus*-infected individuals was previously shown (Fendt et al. 2005), implying that CCL17 which is influenced by Th17 signalling might play a role during filarial infections. Indeed, analysis on day 7 p.i. revealed reduced worm burden in IL-17A-deficient mice implying that IL-17A plays an important role during the migration of L3 larvae through the skin. Thus, future studies should investigate the interplay of Th17/CCL17 signalling and the role for mast cell-dependent vascular permeability which might influence the ability of L3 larvae to migrate and later develop into adult worms using IL-17A/mast cell- and/or IL-17A/CCL17-deficient mice. Moreover, subcutaneous *L. sigmodontis* infection with a defined number of infective larvae and analysis of earlier infection time points between days 0–9 p.i. might decipher the role of IL-17A for parasite clearance during L3 migration into the TC (Ajendra et al. 2016; Karadjian et al. 2017). Indeed, Babayan and colleagues have shown that *L. sigmodontis* develops faster in the presence of IL-5 and eosinophils leading to an earlier release and greater number of microfilaria (Babayan et al. 2010). However, in this study, TC levels of IL-5 as well as IL-10 and IL13 were equal between IL-17A^−/−^ and wildtype C57BL/6 mice (data not shown). Thus, further infection experiments should be performed in IL-17A-deficient BALB/c mice to elucidate the role and interaction of IL-5 and IL-17A.

Since fewer worms reach the TC, infiltration of CD4^+^ T cells, CD4^+^Foxp3^+^ Treg and CD4^+^Rorγt^+^pStat3^+^ Th17 cells was also significantly reduced in IL-17A-deficient mice. Indeed, several studies have shown that lymphocyte populations including Treg and Th17 cells (Katawa et al. 2015; Layland et al. 2015; Babu et al. 2009; Babu and Nutman 2012; Taylor et al. 2005) are important for host immunity against the parasite. In addition, Th17 cells are predominant pro-inflammatory cells secreting IL-17A which induce an array of chemokines and thus attract macrophages and T helper cell populations to promote inflammation against pathogens (Sehrawat and Rouse 2017). Since differentiation of Th17 and Treg cells requires TGF-β, the development of both cell types is linked together and interplay is required to control inflammatory responses (Weaver and Hatton 2009). Several studies have shown that an imbalance of Th17/Treg immune responses influences parasitic pathogenesis (Mbow et al. 2013; Pathak et al. 2015). Therefore, besides reduced worm burden, the lack of IL-17A leads to reduced infiltration of CD4^+^, CD4^+^Foxp3^+^ Treg and Th17 cells into the TC creating an environment which promotes worm’s development leading to longer adult worms. Nevertheless, further studies are required to fully decipher the role of Th17 signalling on worm development and host immunity at the site of infection and especially in the early phase of infection (invasion of L3 larvae in the skin).

Interestingly, neutrophil infiltration into the TC was comparable between infected IL-17A-deficient mice and WT control groups even though IL-17A was previously shown to be important for the recruitment and activation of neutrophils through the induction of a variety of cytokines and chemokines during lung inflammation (Allen et al. 2015). Although the eosinophil recruiting chemokine eotaxin-1 was significantly reduced in the TC of infected IL-17A^−/−^ mice, the number of eosinophils in the TC was comparable between IL-17A^−/−^ and WT groups. This suggests that *L. sigmodontis*-driven recruitment of neutrophils and eosinophils into the TC is independent of IL-17A secretion but may affect their functional responses. Potentially, the lack of IL-17A and reduced eotaxin-1 levels in the TC can be compensated through other neutrophil or eosinophil recruitment/activation chemokines like CXCL1, CXCL5 and CXCL8 (Allen et al. 2015) or MCP-5, MIP-1α, CXCL9, CXCL10, CXCL12 and RANTES (Simon et al. 2004), respectively. Indeed, RANTES levels were increased (not significantly) in a couple of IL-17A^−/−^ mice when compared to WT controls. Since *L. sigmodontis* worms have several developmental states in the host, the immune cell infiltration and thus chemokine/cytokine secretion vary with time. Previous studies have shown that the infiltration of eosinophils and neutrophils into the TC and their attachment to the worms is stage-specific in infected C57BL/6 mice. Moreover, granuloma formation around *L. sigmodontis* worms in BALB/c mice is mainly composed of neutrophils in the later stages of infection (Al-Qaoud et al. 2000; Attout et al. 2008) and RANTES secretion was shown to be more critical for eosinophil recruitment at later time points during allergic airway inflammation (Gonzalo et al. 1998). Since C57BL/6 mice eliminate *L. sigmodontis* worms by day 60 p.i. whereas BALB/c mice provide an environment to patent infection, further *L. sigmodontis* infection experiments analysing different time points of infection in BALB/c mice may provide evidence about the role of IL-17A on microfilaria production and immune cell infiltration as well as cytokine/chemokine levels in the TC.

Today, six IL-17 homologous molecules are known (IL-17A-F) and most of the research is focusing on IL-17A and IL-17F which were shown to be important for autoimmunity and inflammation (Kuwabara et al. 2017). Although IL-17A and IL-17F are highly homologous and share receptors (Gonzalo et al. 1998), distinct roles of these two cytokines have been reported (Ishigame et al. 2009; Yang et al. 2008). Thus, further *L. sigmodontis* infection experiments using IL-17F^−/−^ have to be performed to gain in-depth insight into IL-17 signalling mechanisms during filariasis. In addition to the cytokine and chemokine levels in the TC, we also analysed immune responses derived from draining mLN cells upon LsAg stimulation and observed increased filarial-specific IFN-γ levels in IL-17A-deficient mice. Since IFN-γ secretion upon stimulation with a general T cell receptor activator (αCD3/αCD28) was equal between the infected groups of mice, this indicates that the elevated IFN-γ secretion was filarial-specific. It was shown that IL-17A and IFN-γ derived from T cells can act synergistically to initiate inflammatory responses (Eid et al. 2009), and moreover, immune responses and overt pathology are associated with filarial-specific Th1 and Th17 immune responses (Babu et al. 2009; Anthony et al. 2007; McSorley and Maizels 2012). Thus, the lack of IL-17A could be compensated by Th1-secreting IFN-γ cells leading to increased IFN-γ production upon LsAg stimulation. Further IFN-γ blocking experiments might decipher the connection between IL-17A and IFN-γ during an *L. sigmodontis* infection in more detail.

Overall, these findings show that Th17 signalling, especially IL-17A secretion, is an important player within the complex defence mechanisms of the host but might also be beneficial for the survival of the helminth in the early phases of infection. Again, this highlights the evolutionary evolved dependence and the complexity of the filarial-host interaction.

## Electronic supplementary material

Online Resource 1 Reduced cellular infiltration into the TC of infected IL-17A^−/−^ mice. Groups of WT and IL-17A^−/−^ mice were infected with *L. sigmodontis* for 28 days. Thereafter, cells within the TC (a) or mLN (b) were determined. Values are expressed as mean ± SEM and symbols show levels in each mouse from 4 independent infection experiments (*n* = 20 IL-17A^−/−^ and *n* = 23 WT mice). Statistical significances between the indicated groups were obtained using the unpaired t test (a) and the Mann-Whitney-U-tests (b). Asterisks denote significant differences between the groups indicated by the brackets (*p < 0.05). (PNG 30 kb)

High resolution image (TIF 203 kb)

Online Resource 2 Gating strategy for monocytes, macrophages, neutrophils and eosinophils in the TC. Groups of WT and IL-17A^−/−^ C57BL/6 mice were infected with *L. sigmodontis* for 28 days. TC cells were stained with fluorophore-conjugated anti-mouse CD11b, SiglecF, F4/80, GR1 and Ly6c monoclonal antibodies and frequencies of CD11b^+^SiglecF^+^ eosinophils, CD11b^+^SiglecF^−^Ly6c^+^ monocytes, CD11b^+^SiglecF^−^GR1^+^ neutrophils and CD11b^+^SiglecF^−^F4/80^+^ macrophages were analysed according to the presented gating strategy. (PNG 47 kb)

High resolution image (TIF 301 kb)

Online Resource 3 Peripheral cell differentiation is unchanged in *L. sigmodontis*-infected IL-17A^−/−^ C57BL/6 mice. Groups of WT and IL-17A^−/−^ C57BL/6 mice were infected with *L. sigmodontis* for 28 days. In peripheral blood, the frequency of macrophages (a), lymphocytes (b), neutrophils (c) and eosinophils (d) were determined in individual mice using microscopy. Values are expressed as mean ± SEM and symbols show levels in each mouse from 3 independent infection experiments (*n* = 10 IL-17A^−/−^ and *n* = 13 WT mice). Statistical significances between the indicated groups were obtained using the Mann-Whitney-U-tests. (PNG 799 kb)

High resolution image (TIF 326 kb)

Online Resource 4 Gating strategy for CD4^+^ and CD4^+^Foxp3^+^ cell populations. Groups of WT and IL-17A^−/−^ C57BL/6 mice were infected with *L. sigmodontis* for 28 days. TC and mLN cells were stained with fluorophore-conjugated anti-mouse CD4 and Foxp3 monoclonal antibodies and frequencies of CD4^+^ T cells and CD4^+^Foxp3^+^ Treg were analysed according to the presented gating strategy. (PNG 158 kb)

Online Resource 5 Gating strategy for CD4^+^Rorγt^+^pStat3^+^ and CD4^+^Rorγt^+^pStat3^+^IL-17A^+^ cell populations. Groups of WT and IL-17A^−/−^ C57BL/6 mice were infected with *L. sigmodontis* for 28 days. TC and mLN cells were stained with fluorophore-conjugated anti-mouse CD4, Rorγt^,^ pStat3 (Y705) and IL-17A monoclonal antibodies and frequencies of CD4^+^Rorγt^+^pStat3^+^ and CD4^+^Rorγt^+^pStat3^+^IL-17A^+^ Th17 cells were analysed according to the presented gating strategy. (PNG 199 kb)

## Abbreviations

Cont.: unstimulated cell cultures
LF: lymphatic filariasis
*L. sigmodontis*: *Litomosoides sigmodontis*
LsAg: *L. sigmodontis* worm antigen preparation
mLN: mediastinal lymph nodes
p.i.: post-infection
RANTES: regulated on activation, normal T cell expressed and secreted
TC: thoracic cavity
Treg: regulatory T cells
WT: wildtype
