# Supplementary figures and images for: Absence of IL-17A in Litomosoides sigmodontis-infected mice influences worm development and drives elevated filarial-specific IFN-γ
Source: Parasitol Res. 2018 Jun 22;117(8):2665–75. doi: 10.1007/s00436-018-5959-7 (PMC6061040; doi:10.1007/s00436-018-5959-7)

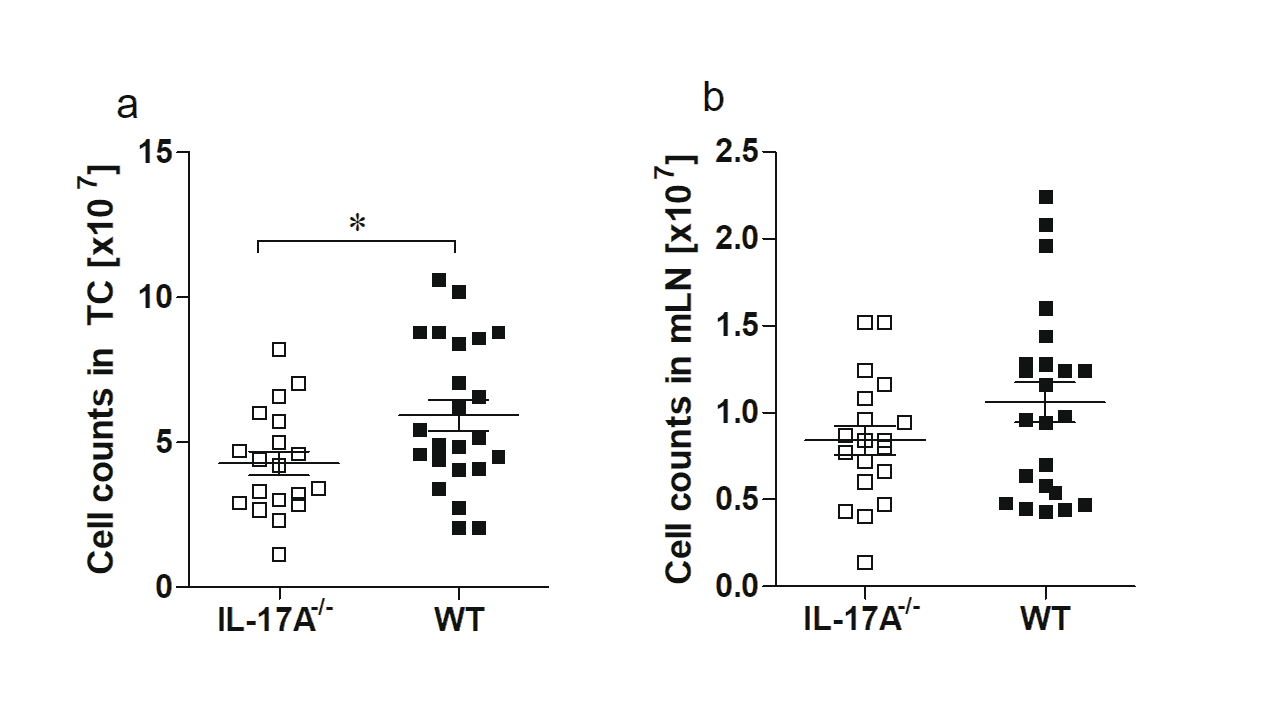

Supplement: Supplementary file 1 — Reduced cellular infiltration into the TC of infected IL-17A−/− mice. Groups of WT and IL-17A−/− mice were infected with L. sigmodontis for 28 days. Thereafter, cells within the TC (a) or mLN (b) were determined. Values are expressed as mean ± SEM and symbols show levels in each mouse from 4 independent infection experiments (n = 20 IL-17A−/− and n = 23 WT mice). Statistical significances between the indicated groups were obtained using the unpaired t test (a) and the Mann-Whitney-U-tests (b). Asterisks denote significant differences between the groups indicated by the brackets (*p < 0.05). (PNG 30 kb) [file 436_2018_5959_Fig7_ESM.png]

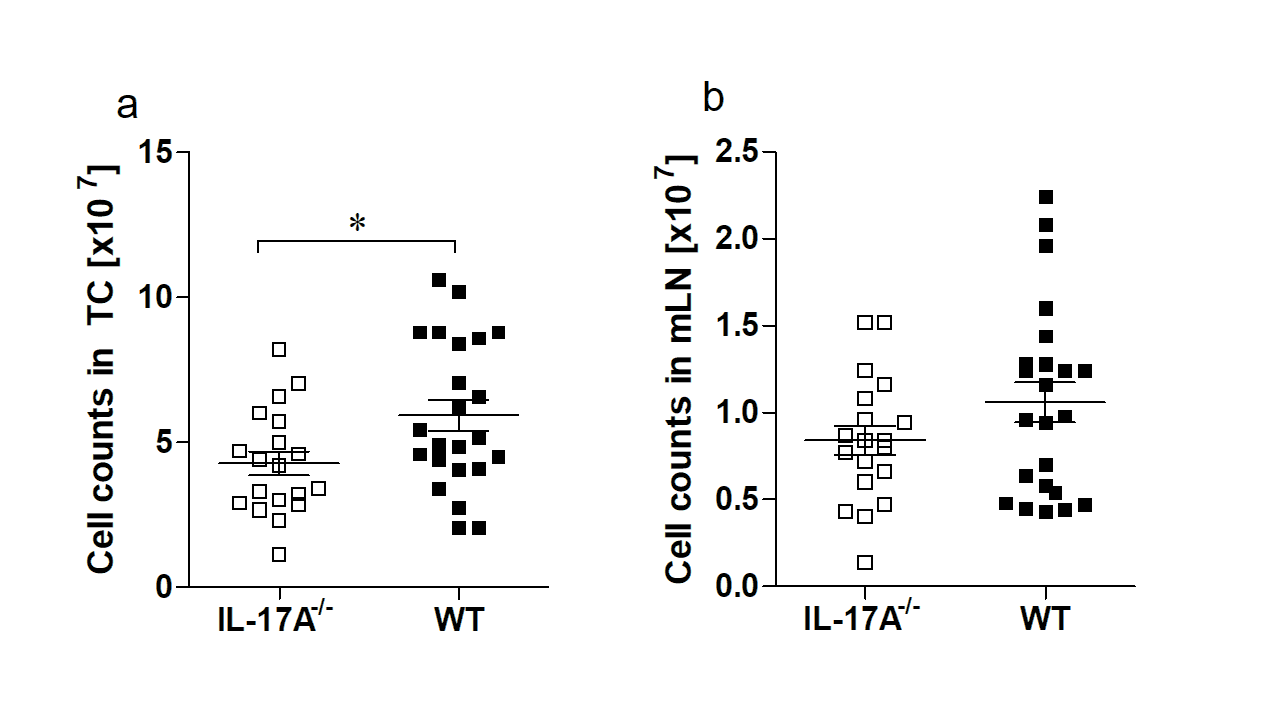

Supplement: Supplementary file 2 — High resolution image (TIF 203 kb) [file 436_2018_5959_MOESM1_ESM.tif]

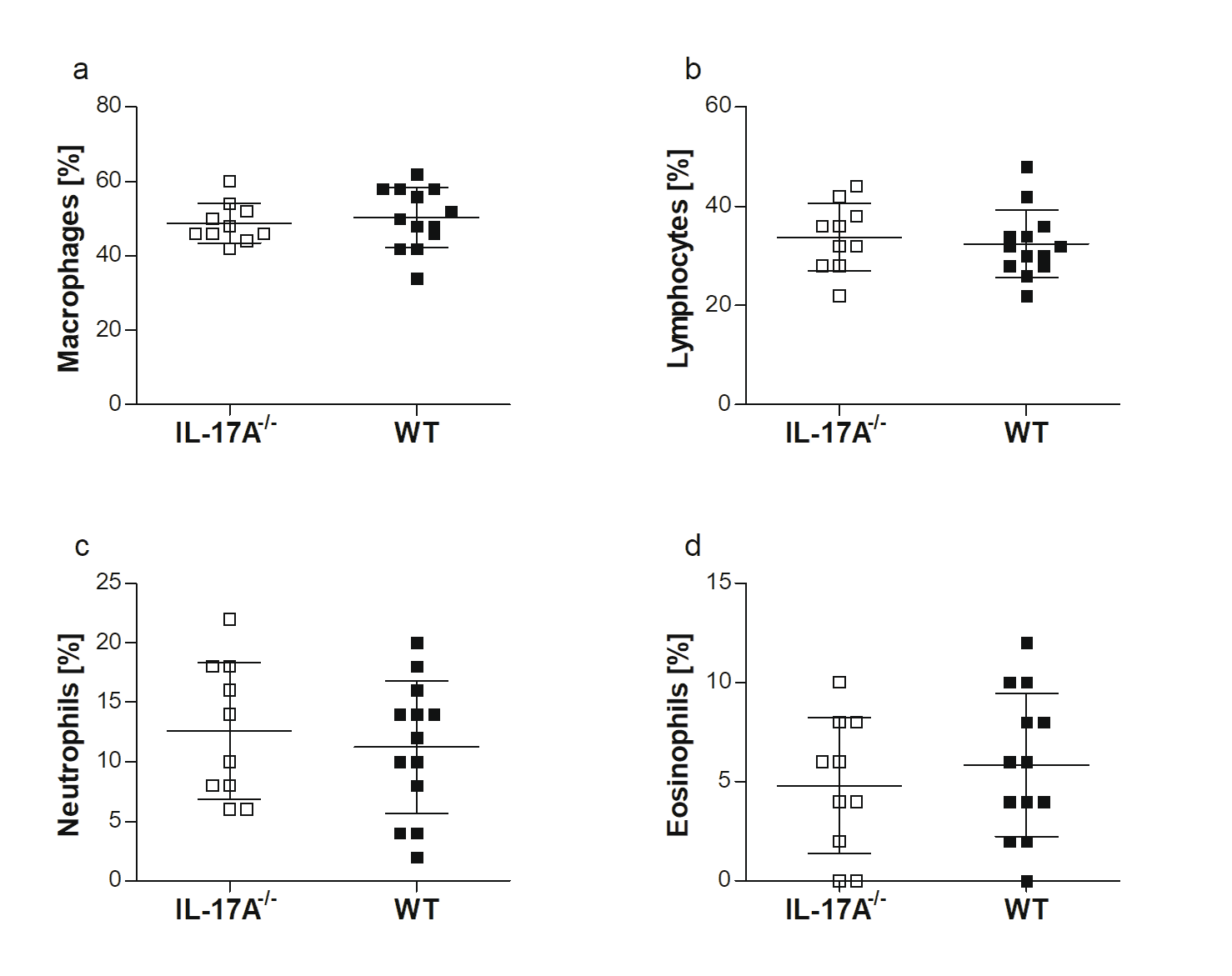

Supplement: Supplementary file 3 — Gating strategy for monocytes, macrophages, neutrophils and eosinophils in the TC. Groups of WT and IL-17A−/− C57BL/6 mice were infected with L. sigmodontis for 28 days. TC cells were stained with fluorophore-conjugated anti-mouse CD11b, SiglecF, F4/80, GR1 and Ly6c monoclonal antibodies and frequencies of CD11b+SiglecF+ eosinophils, CD11b+SiglecF−Ly6c+ monocytes, CD11b+SiglecF−GR1+ neutrophils and CD11b+SiglecF−F4/80+ macrophages were analysed according to the presented gating strategy. (PNG 47 kb) [file 436_2018_5959_Fig8_ESM.png]

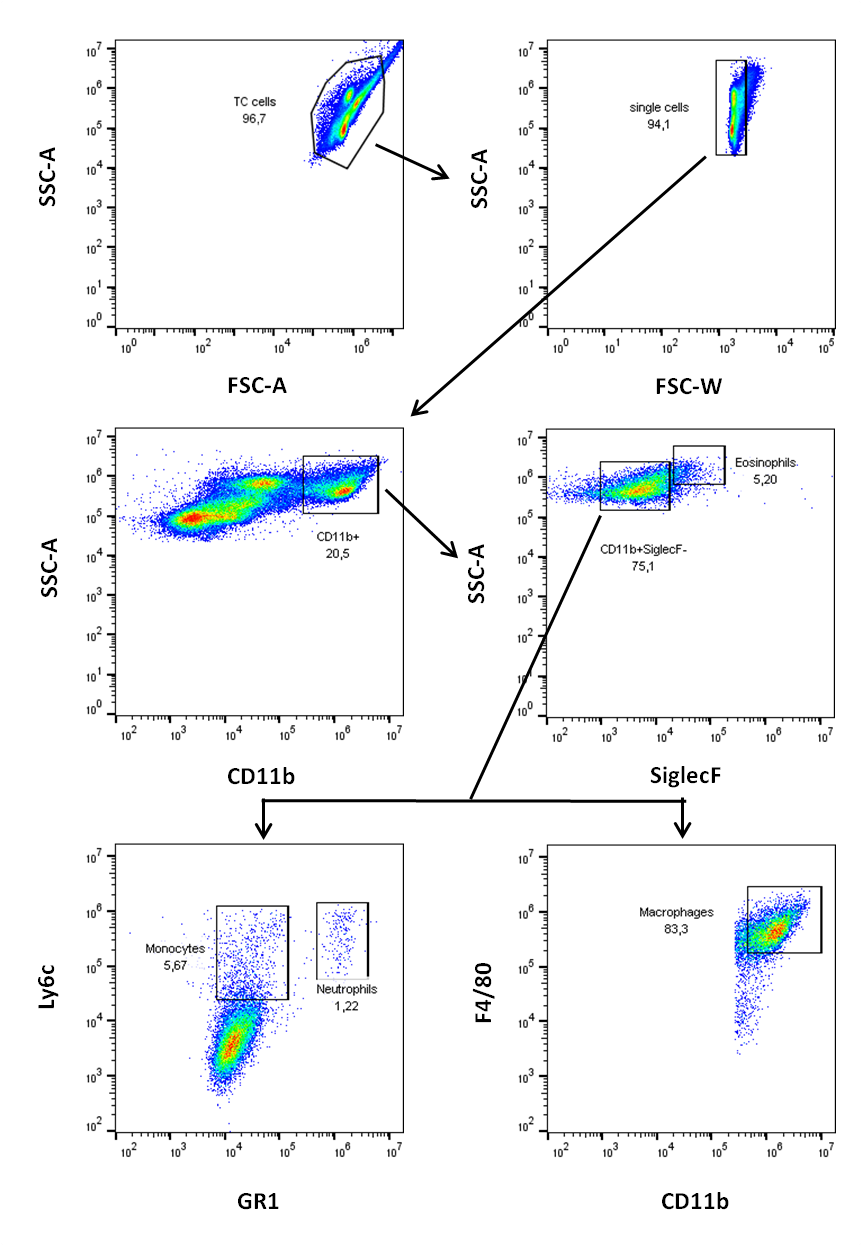

Supplement: Supplementary file 4 — High resolution image (TIF 301 kb) [file 436_2018_5959_MOESM2_ESM.tif]

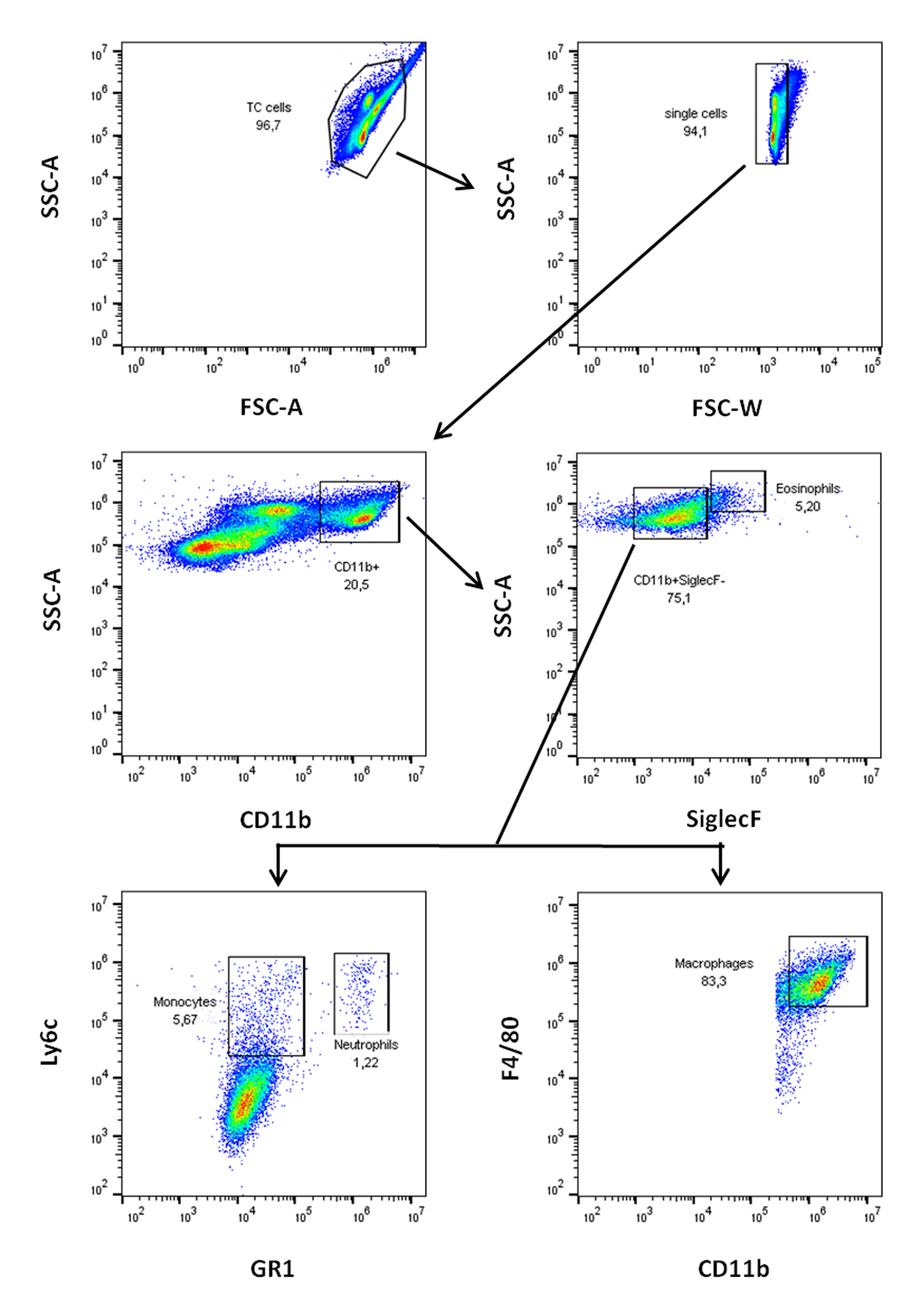

Supplement: Supplementary file 5 — Peripheral cell differentiation is unchanged in L. sigmodontis-infected IL-17A−/− C57BL/6 mice. Groups of WT and IL-17A−/− C57BL/6 mice were infected with L. sigmodontis for 28 days. In peripheral blood, the frequency of macrophages (a), lymphocytes (b), neutrophils (c) and eosinophils (d) were determined in individual mice using microscopy. Values are expressed as mean ± SEM and symbols show levels in each mouse from 3 independent infection experiments (n = 10 IL-17A−/− and n = 13 WT mice). Statistical significances between the indicated groups were obtained using the Mann-Whitney-U-tests. (PNG 799 kb) [file 436_2018_5959_Fig9_ESM.png]

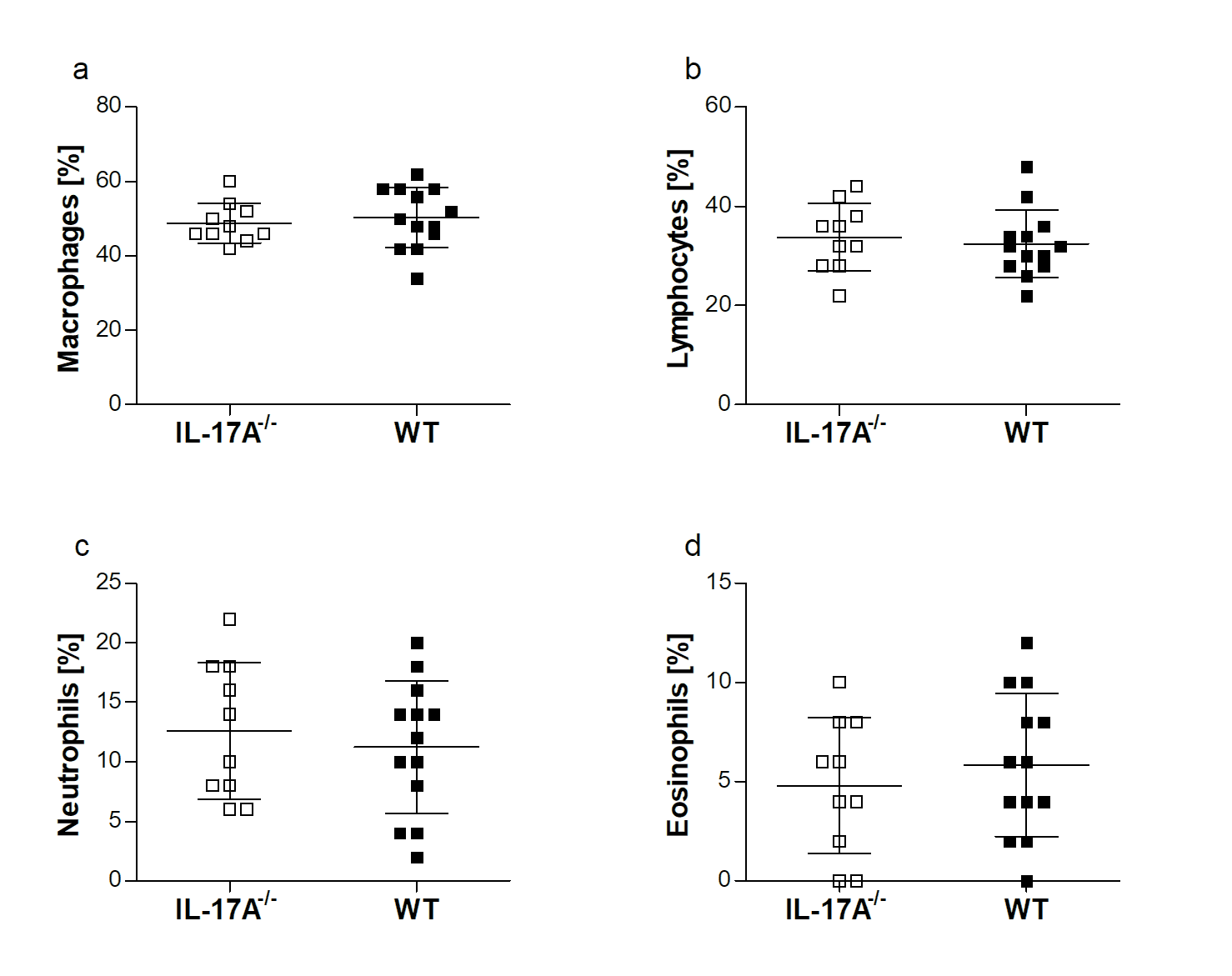

Supplement: Supplementary file 6 — High resolution image (TIF 326 kb) [file 436_2018_5959_MOESM3_ESM.tif]

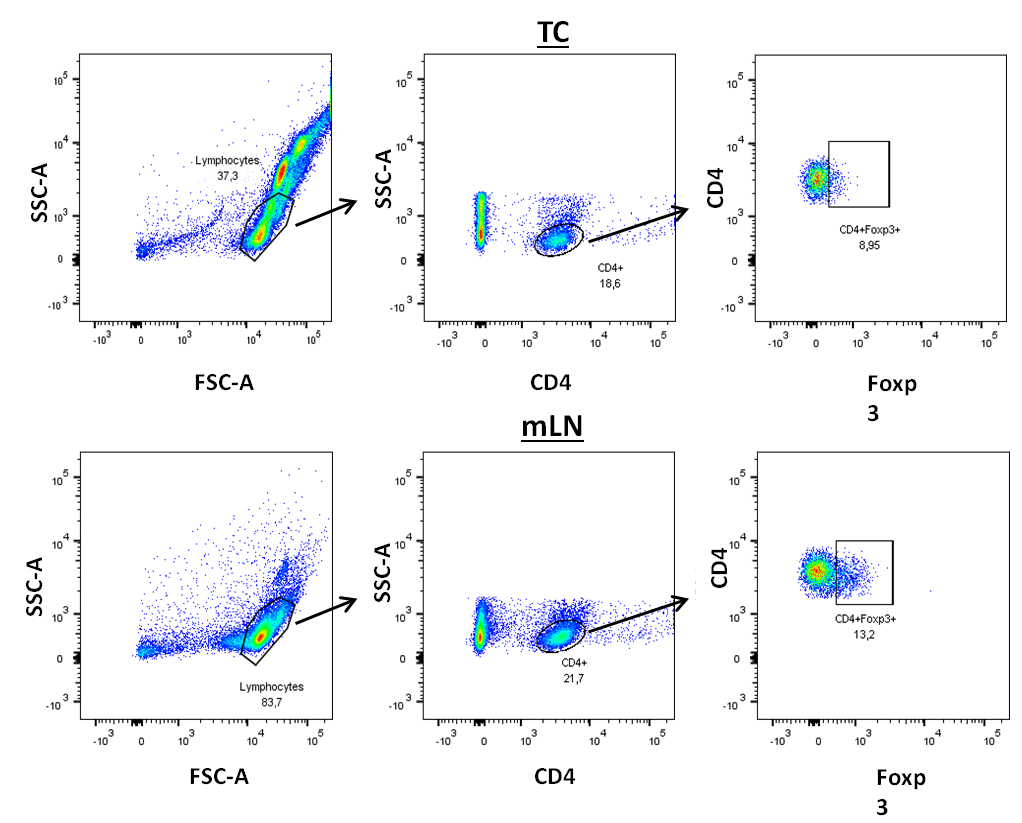

Supplement: Supplementary file 7 — Gating strategy for CD4+ and CD4+Foxp3+ cell populations. Groups of WT and IL-17A−/− C57BL/6 mice were infected with L. sigmodontis for 28 days. TC and mLN cells were stained with fluorophore-conjugated anti-mouse CD4 and Foxp3 monoclonal antibodies and frequencies of CD4+ T cells and CD4+Foxp3+ Treg were analysed according to the presented gating strategy. (PNG 158 kb) [file 436_2018_5959_MOESM4_ESM.png]

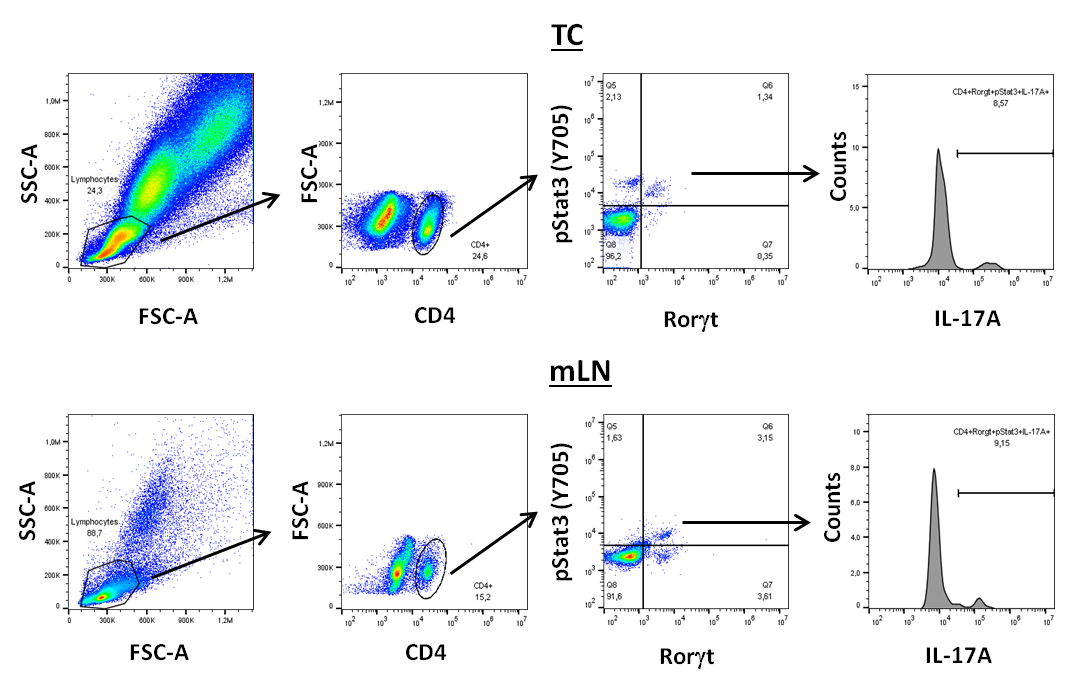

Supplement: Supplementary file 8 — Gating strategy for CD4+Rorγt+pStat3+ and CD4+Rorγt+pStat3+IL-17A+ cell populations. Groups of WT and IL-17A−/− C57BL/6 mice were infected with L. sigmodontis for 28 days. TC and mLN cells were stained with fluorophore-conjugated anti-mouse CD4, Rorγt, pStat3 (Y705) and IL-17A monoclonal antibodies and frequencies of CD4+Rorγt+pStat3+ and CD4+Rorγt+pStat3+IL-17A+ Th17 cells were analysed according to the presented gating strategy. (PNG 199 kb) [file 436_2018_5959_MOESM5_ESM.png]
